# Annotations

**Published:** 1903-11-21

**Authors:** 


### ANNOTATIONS.

### The Kaiser.

The intelligence that it was found necessary, last week, to remove what was described as a "polypus" from the larynx of the German Emperor was naturally received with something like consternation throughout his dominions, and with very widespread regret in other parts of the world. The apparent gravity of the case, however, seems to be almost entirely due to the family history of the illustrious patient, and to the fact that the locality of the growth is that in which the disease fatal to his father was first detected. Fortunately, however, it cannot be argued that an inherited liability to malignant growths, even supposing it to exist, confers any immunity from those which are benign, or affords anything which can be called evidence as to their nature. As far as the examination of the removed tissue is concerned, the testimony of Professor Orth as to the results of his microscopical examination is entirely satisfactory, and shows that no single characteristic of malignancy could be detected. Notwithstanding this, it will be impossible for the public anxiety of Germany to be allayed until the correctness of the diagnosis has been established by the lapse of time; for surgeons are only too familiar with the fact that growths, at first apparently of simple character, may in time recur and assume the character of malignancy. It will, of course, be impossible that the truth as to such matters should be withheld from the Kaiser. In these circumstances he will be called upon to display that courage of passive endurance, and that power to sustain suspense, which are perhaps even greater than the courage which his ancestors have displayed in fields of action. His avowed determination to conceal nothing is worthy of the place which he occupies in the eyes of Europe and of the world, and will add to the solicitude with which all further information as to his condition will be awaited. What is so far known fully justifies hope, but can hardly be regarded as affording certainty.

### Wise and Enlightened Governors.

In addressing the Lord Mayor on behalf of the deputation of Governors of St. Bartholomew's Hospital on Tuesday last, Sir Trevor Lawrence again called attention to the fact that the Lord Mayor's committee had affirmed "that the reputation and character of the hospital had been fully vindicated, and that its administration had been conducted in a wise and enlightened spirit, with due regard to economy and the best interests of the patients." Now it is not quite easy to understand exactly how this statement can be reconciled with the remarks made by two of the consulting surgeons of the hospital at the recent meeting of the General Court of Governors, which were to the effect that "the out-patient department was in a disgusting state, unfit for human beings," and that "its condition was nothing short of a scandal." If such a state of affairs cannot be avoided even under wise and enlightened management our hospital system itself must be at fault. Again, the reason which has caused the Governors to reject the good advice of the Medical Council—the advice, that is, of the highest experts in the land, whose life-work has been intimately connected with St. Bartholomew's Hospital—appears to be that sufficient money would not be forthcoming from the public to enable them to do that which is right and proper. If this, again, be true, our voluntary hospital system stands condemned. As a nation we are justly proud of this system, founded as it is in the hearts of the people, as the noblest institution of our age; and the question of the future of St. Bartholomew's Hospital, therefore, at once becomes one of national importance. In face of these facts we feel sure that the City of London and the general public will insist on a proper, adequate, and thorough reconstruction of the hospital on a larger site than it now possesses, and that when such a plan is placed before them they will rise to the occasion, as they have done in the past, and see to it that a sufficient sum of money is provided to carry the scheme into effect.

### The Temperature in Cases of Coma.

The state of the temperature in cases of coma is a matter of some moment, as it is one of the factors influencing diagnosis. When the coma depends on hæmorrhage into the pons, a rapidly developing high temperature is one of the ruling facts and materially assists in the interpretation of the case. In uræmic and diabetic coma, in poisoning by opium, and in concussion of the brain, on the other hand, the temperature tends to be subnormal. Probably the question in the great majority of cases lies between an ordinary (not pontine) hæmorrhage and poisoning by alcohol, and it is here that authoritative statements regarding the temperature betray some measure of contradiction. The late Sir Benjamin Ward Richardson used to teach that in apoplexy the temperature is raised, whilst in alcoholic coma it is subnormal. Nowadays it will be generally allowed that the terms of this proposition require qualification. It is beyond question that the temperature immediately after the onset of an attack of apoplexy may be below normal. Later, the blood-clot presumably acting as a foreign body and causing irritation, there may be appreciable febrile disturbance. As regards alcoholic poisoning modern statements are somewhat wanting in precision. There can be no doubt that in the majority of the "dead drunks" who are carried to a police station the temperature is depressed. The conditions afford a natural explanation of this. Most of these patients are found after they have been lying for some hours exposed to the elements and often unprotected by adequate clothing. That is, the circumstances are such as favour loss of heat, the chance for this to occur being all the greater in consequence of the dilating influence of the poison on the cutaneous vessels. But in circumstances which do not establish these conditions alcoholic coma is certainly not always attended by a low thermometric record. Thus a patient found unconscious in his bed as the result of a large dose of alcohol may show a normal or even a febrile temperature. This at least is an occasional experience, and it would be well if those who record instances of acute poisoning by alcohol would add a note of the temperature in relation to the conditions in which the patient was situated.

